# My Love–Hate Relationship with *Loxosceles* in Africa

**DOI:** 10.4269/ajtmh.20-0825

**Published:** 2020-11

**Authors:** Julie M. Buser

**Affiliations:** Fogarty Global Health Fellow, Fogarty International Center, National Institutes of Health, Global Reach, University of Michigan Medical School, Ann Arbor, Michigan

The rhythmic sound of ocean waves served as a lullaby that fateful night. While I lay peacefully sleeping in an eco-resort near Accra on Ghana’s picturesque Atlantic Coast, an itsy bitsy spider bit me. When it happened, I did not even wake up. In the morning, after a hearty traditional Ghanaian breakfast of waakye (red beans and rice), I caught myself itching the back of my left thigh. Because there was just a tiny speck of redness, I brushed off the irritation as yet another annoying mosquito bite. I am extremely vigilant about taking antimalarial prophylaxis and presumed I would be alright. I had traveled from the Ashanti region, where I was doing a postdoctoral research fellowship in global health, to Accra so I would be closer to the capital’s international airport. I looked forward to catching a flight to Rwanda to attend an international conference for women in global health leadership.

In Rwanda, I started experiencing some localized pain but not much swelling. I stopped by the conference center clinic for a consultation in between sessions and was told not to worry about my leg because there were no signs of infection. Over the next couple of days, as fatigue and body aches set in, I noticed a couple of small puncture marks and increasing erythema. Sadly, I did not feel much like networking with conference participants. I thought I would feel better after a couple of days’ rest during my planned visit to Lake Kivu.

By the time I got to Gisenyi, a town near the lake, a week had passed, and the bite started to ulcerate. I knew it was time to seek medical attention again. With the help of a host from my youth hostel, I visited a Congolese doctor in a private clinic who prescribed oral antibiotics and daily dressing changes along with a pommade ichtyolée. I spoke just enough French and he spoke just enough English that we could communicate a plan of care. I was relieved to have a course of action and thought I would be on the mend by the time I returned to Ghana the following day.

I was wrong. Necrosis started to set in. The wound was small but looked dangerous. I landed back in Accra and went straight to a private hospital. The first doctor I saw in the outpatient clinic sent me to the emergency room where I was consulted by a surgeon who diagnosed me with a *Loxosceles* spider bite and admitted me overnight for IV antibiotics, dressing changes, and antihistamines.

The next stage of my journey began as an exceedingly thin orderly helped me into a wheelchair. For living in such a humid tropical environment, he had surprisingly cool hands. As he wheeled me to the ward, the moans of other patients, combined with the horn-blaring and shouting of taxi drivers from the frenzied city streets, created a cacophony of sounds. Windows and doors were left open throughout the hospital, allowing noise to travel freely. I could not help but notice the juxtaposition with the hospital in which I work in Michigan, where designated quiet hours were created for patients to rest. As we traveled down a labyrinth of long hallways, I shut my eyes against the intense stares of patients and caregivers straining to observe the oburoni, or foreigner, pass by. En route, the orderly openly shared my reason for being admitted with anyone who inquired. I perceived a definite lack of patient privacy.

Thankfully, by the next morning, my leg improved enough after a couple of doses of IV antibiotics, and I was discharged on oral medications with daily dressing changes. Finally, I could breathe more easily knowing I was on the mend and that nasty little *Loxosceles* spider would not get the best of me. Oh, how I hated that particular spider!

My recovery process was slow. Because of the location of the bite, I could not reach the wound to properly clean and apply medication. I made daily trips to various public and private clinics for dressing changes. Over the next 5–6 weeks, the wound healed by secondary intention. Today, I am left with just a tiny scar and unforgettable memories of being on the receiving end of clinical tropical medicine and health care in two African countries.

Until my run in with *Loxosceles*, my experience with health care in Africa was from the perspective of provider and educator. In the past, I worked as a health project coordinator and advanced practice nurse. Providing medical humanitarian assistance in various sub-Saharan Africa countries was an enlightening experience for me. I learned to approach life with an open-mind and open-heart through the development of an understanding of other cultures and ways of life. I continued an exploration of other cultures while conducting doctoral research in rural Zambia and postdoctoral research in urban Ghana.

Thanks to that *Loxosceles* spider, I was exposed to the patient perspective of health care in Africa. As much as I struggled with the ramifications of the dreadful spider bite, I developed an appreciation for the mysterious *Loxosceles*. Scared and alone, I had to be my own patient advocate and was humbled by the challenges of navigating an unfamiliar health system. I am grateful to the spider for being the spark that helped me gain new insight into health care in Africa. I learned what it is like to hobble back and forth from the cashier’s office to pay for supplies every time I needed an IV catheter or new medication. I recognized the need for improved sanitation and hygiene as I waited for dressing changes in questionable environments. I was reminded of how important it is for patients to be patient as I faced dressing supply stock outages and challenges filling prescriptions at pharmacies. I understood the shortage of health workers as never before when no nurses checked on me for 7 hours overnight.

My experience as a patient reinforced my interest in improving global health. Although I hate *Loxosceles* for causing me distress, I love the spider for exposing me to the patient side of health care in Africa. As part of my self-reflection and practice of cultural humility, I remind myself to acknowledge power imbalances and the importance of developing global partnerships. Undoubtedly, Americans can learn from Rwanda’s emphasis on health equity, use of community health workers, and community-based health insurance. We can also glean important lessons from Ghana’s efforts to provide health care for all of its citizens with a universal health insurance scheme.

In this Year of the Nurse and the Midwife, through my encounter with *Loxosceles*, I solidified my goal as a PhD nurse-scientist to positively contribute to the disciplines of tropical medicine and global health by performing revolutionary scientific research and teaching. I now have a better, firsthand understanding of the need to improve access to care and promote sustainable health infrastructure in sub-Saharan Africa. I intend to take full advantage of the lessons learned from my love–hate relationship with *Loxosceles* to generate new knowledge and develop relationships to explore innovative ways of improving global health.

